# Asynchronous parallel Bayesian optimization for AI-driven cloud laboratories

**DOI:** 10.1093/bioinformatics/btab291

**Published:** 2021-07-12

**Authors:** Trevor S Frisby, Zhiyun Gong, Christopher James Langmead

**Affiliations:** Computational Biology Department, School of Computer Science, Carnegie Mellon University, Pittsburgh, PA 15213, USA; Computational Biology Department, School of Computer Science, Carnegie Mellon University, Pittsburgh, PA 15213, USA; Computational Biology Department, School of Computer Science, Carnegie Mellon University, Pittsburgh, PA 15213, USA

## Abstract

**Motivation:**

The recent emergence of cloud laboratories—collections of automated wet-lab instruments that are accessed remotely, presents new opportunities to apply Artificial Intelligence and Machine Learning in scientific research. Among these is the challenge of automating the process of optimizing experimental protocols to maximize data quality.

**Results:**

We introduce a new deterministic algorithm, called **P**a**R**allel **O**ptimiza**T**i**O**n for **C**l**O**ud **L**aboratories (PROTOCOL), that improves experimental protocols via asynchronous, parallel Bayesian optimization. The algorithm achieves exponential convergence with respect to simple regret. We demonstrate PROTOCOL in both simulated and real-world cloud labs. In the simulated lab, it outperforms alternative approaches to Bayesian optimization in terms of its ability to find optimal configurations, and the number of experiments required to find the optimum. In the real-world lab, the algorithm makes progress toward the optimal setting.

**Data availability and implementation:**

PROTOCOL is available as both a stand-alone Python library, and as part of a R Shiny application at https://github.com/clangmead/PROTOCOL. Data are available at the same repository.

**Supplementary information:**

Supplementary data are available at *Bioinformatics* online.

## 1 Introduction

Most standard laboratory techniques have been automated which, in turn, has enabled the development of commercial robotic *Cloud Laboratories* (e.g. EmeraldCloudLab and Transcriptic), and the emergence of a new paradigm of *Science-as-a-Service*. Analogous to cloud computing, cloud labs let scientists outsource the management and maintenance of a set of resources—automated scientific instruments, and thus devote more time and money to their research. In addition to these administrative and economic benefits, cloud labs also significantly increase the reproducibility of scientific research, due to the use of robotics. For these reasons, one can anticipate an increase in the utilization of cloud labs by scientists in academia and industry alike, at least for certain tasks.

Existing cloud labs are largely open-loop, in the sense that humans must specify every detail of the experimental protocols to be executed by the robots. However, it is not difficult to imagine an AI-driven cloud lab that automatically finds optimal instrument settings and/or experimental conditions, so as to maximize throughput and data quality, or to minimize costs. Eventually, such systems might lead to the widespread use of general-purpose ‘robot scientists’ capable of making novel discoveries autonomously, as first demonstrated in 2004 (King *et al.*, 2004). Toward these ends, this paper introduces a method, called **P**a**R**allel **O**ptimiza**T**i**O**n for **C**l**O**ud **L**aboratories (PROTOCOL), to perform closed-loop optimization of experimental protocols against a user-defined objective.

PROTOCOL builds on recent work in Bayesian Optimization (BO) (Snoek *et al.*, 2012; Mockus, 1989), which is a sequential strategy for optimizing black-box (i.e. unknown) functions. The technique is Bayesian because it places a prior distribution over the objective function, and then computes posteriors at the end of each round, based on the outcome of an algorithmically selected function evaluation. The key differences between BO methods are the means by which they represent the distribution over functions, and the way that they select the next design configuration to test. Gaussian Processes are a very common choice for specifying the distribution, and that is what is used in this paper. The selection strategy, sometimes called the *acquisition function*, will define a utility function and then searches for a design with (approximately) maximal utility. PROTOCOL introduces a novel acquisition strategy that is matched to the features of a cloud lab environment.

Bayesian Optimization is often used in application domains where the evaluation of the objective function is extremely slow or expensive, such as hyperparameter optimization in deep learning (e.g. Bergstra *et al.*, 2011). Performing wet-lab experiments is also time-consuming, even under the best of circumstances. But cloud labs comprise a set of shared resources, and so experiments often sit in a queue waiting for specific instruments to become available. That is, the benefits of outsourcing the management and maintenance of a wet-lab to the cloud are somewhat offset by increased cycle times, on a per-experiment basis. On the other hand, a suitably equipped cloud lab may facilitate *parallel searches* for optimal conditions. PROTOCOL’s acquisition function takes advantage of such parallelism by selecting *batches* of designs to test, while performing closed-loop, asynchronous Bayesian Optimization.

We evaluated PROTOCOL on two test scenarios. The first optimized four instrument parameters for MALDI-ToF mass spectrometry in a simulated cloud lab (but using real data). The second optimized five instrument parameters *and* the solvent ratio for HPLC in a real cloud lab. PROTOCOL outperforms conventional BO methods dramatically on the MALDI-ToF data, given the same budget. On the real cloud lab, PROTOCOL makes progress toward finding a high-resolution chromatogram.

## 2 Background and related work

### 2.1 Bayesian optimization with Gaussian processes

Bayesian Optimization is a sequential strategy for optimizing black-box objective functions, *f*. As mentioned in the introduction, Gaussian Processes (GP) are frequently used to represent and compute over the distribution *P*(*f*). A GP is defined by a mean function μ:X→R and kernel function K:X×X→R. Common choices for *K* include radial basis function (RBF) and Matérn kernels. In a typical application of GP-based BO, it is assumed that f ∼ GP(μ,K), and thus f(x) ∼ N(μ(x),K(x,x′)), for all x∈X. This is useful because it provides a straightforward way to obtain closed-form posterior mean ($\mu_{P}$) and variance functions:

(1) $$\begin{array}{c} \mu_{P}(x)=E[f(x)]=K_{x,x\prime }{\left(K+\sigma^{2}I\right)}^{-1}y\\ \text{Var}[f(x)]=K_{x,x}-K_{x,x\prime }{\left(K+\sigma^{2}I\right)}^{-1}K_{x\prime ,x} \end{array}$$

Together, these functions can be used to compute posterior probabilities over function values, and related quantities, such as upper and lower confidence values. These estimates can be used by an acquisition function to identify untested points (i.e. designs) that will provide information relevant to finding the optimal value of *f*.

### 2.2 Bound-based Bayesian optimization

Various acquisition function strategies have been proposed, including Thompson Sampling (TS) (Agrawal and Goyal, 2012), Expected Improvement (EI), Probability of Improvement (PI) and Upper Confidence Bounds (UCB) (Wilson *et al.*, 2018). A common feature of these methods—and a potential weakness, is that they require access to a finite sampling procedure which affects both the runtime and the ultimate resolution the optimization procedure—the finer the resolution, the more computationally expensive the optimization. Recently, however, an algorithm that does not require sampling during BO was introduced. That algorithm, called IMGPO (Infinite Metric Gaussian Process Optimization) (Kawaguchi *et al.*, 2015), performs serial BO and comes with convergence guarantees. Our method extends IGMPO to the asynchronous parallel setting.

IMGPO builds upon previous work in bound-based optimization methods (Munos, 2011; Wang *et al.*, 2014), and uses a divide-and-search strategy based on estimated bounds, like DIRECT (Jones *et al.*, 1993). It proceeds by growing a hierarchical partitioning tree over an *n*-dimensional search space while maintaining a GP model conditioned on observations previously requested by the algorithm. The tree is grown by iterating over it in a top-down fashion and choosing whether or not to evaluate the center of intervals/hyperrectangles associated with each node in the tree. Selected intervals may be further divided into three subintervals along the hyperrectangle’s longest dimension, resulting in three new leaf nodes in the tree.

The decision to evaluate and divide is made by comparing multiple bounds on the unknown ground-truth value of a given interval’s center. These bounds are defined by the upper confidence bounds (UCB) of the GP model (In this article, we assume that we are trying to maximize the objective function. If attempting to minimize a function, one uses lower confidence bounds (LCB).), as well as the ground-truth values of observed interval centers. In general, when the UCB of the current iterate is greater than the current best observed value, the algorithm will request to evaluate the current interval’s center, and divide the interval. Ultimately, the sequence of selected interval centers that are evaluated converge to the ground-truth function’s optimal value. The IMGPO authors prove that their algorithm achieves exponential convergence over continuous search spaces with respect to simple regret, given by $R(x^{+})=sup_{x\in X}f(x)-f(x^{+})$, where *x*^+^ is the configuration found by the algorithm, without the need for sampling of the input space. For full technical details of the algorithm and proofs, we refer the reader to their paper (Kawaguchi *et al.*, 2015). We emphasize that the IMGPO algorithm performs serial optimization, in that it only requests the evaluation of one design at a time.

## 3 Our method: PROTOCOL

PROTOCOL adopts the hierarchical partitioning tree schema and interval division criteria employed by IMGPO. The primary innovation used by PROTOCOL is the calculation of a *frontier* from which up to *k* experiments can be chosen to run in parallel. Here, *k* is the maximum number of experiments a given cloud lab user is authorized to run at the same time. The frontier consists of the center points of the set of *potentially optimal hyperrectangles* in the *n*-dimensional search space (i.e. those that may contain the optimum of the objective function). The idea of maintaining a set of potentially optimal hyperrectangles is borrowed and adapted from the DIRECT algorithm (Jones *et al.*, 1993) for derivative-free global, serial (non-Bayesian) optimization. One of the points on the frontier will always be the point that IMPGO would have selected (in the serial optimization setting), given access to the same set of observations of the objective function. Hence, PROTOCOL inherits the same guarantees as IMGPO, with respect to exponential convergence (see below).

The remaining points on the frontier are identified by computing the convex hull over a two-dimensional encoding of the sub-volumes associated with all non-evaluated leaf nodes in the partition tree. The two coordinates for each sub-volume are the corresponding node’s depth in the tree (which is inversely proportional to the size of the sub-volume), and the UCB of the objective function within that region. The intuition behind maintaining a frontier based on sub-volumes of different sizes is that those volumes represent different trade-offs between *exploration* of the input space—to gather information from under-sampled regions (i.e. those corresponding to relatively large volumes), and *exploitation—*to search in the vicinity of the best design observed thus far (i.e. those corresponding to relatively small volumes). Every BO acquisition function makes a trade-off between exploration and exploitation; PROTOCOL’s strategy is to select batches of experiments that individually make different trade-offs. The use of the UCB is justified based on the well-established principle of optimism under uncertainty (Bubeck and Cesa-Bianchi, 2012). Using the convex hull ensures the algorithm avoids requesting experiments the GP model believes to be suboptimal, while considering nodes at each depth promotes choosing intervals representing varied portions of the input space.

The selection strategy used by PROTOCOL can be described as a three step process:

1. **Identify intervals eligible for division**. This step follows an anologous one in IMGPO, where the algorithm traverses the tree and attempts to identify one interval *at each depth* of the tree that is eligible to be divided. If a function evaluation is necessary, PROTOCOL will calculate a frontier, and multiple experiments may be requested accordingly.
2. **Prune the chosen intervals**. This step also follows directly from IMGPO. Intervals selected in Step 1 are added to a list if they contain a UCB greater than the value associated with any smaller interval in the tree. Essentially, this determines whether or not progress made in other portions of the tree suggests the algorithm should continue to divide in that area or elsewhere.
3. **Select and divide intervals**. This step differs from IMGPO. Here, any intervals that passed the initial two steps are divided into three subintervals by splitting along the longest dimension. Any newly created intervals whose center would be evaluated according to IMGPO are added to an experimental queue. From the remaining experiments, a frontier is calculated, and the queue is filled up to the maximum level of parallelization with experiments that lie on the frontier. In the event that there are more experiments on the frontier than space in the queue, those with highest UCB are chosen. In the event that there aren’t enough intervals to fill the queue (i.e. the size of the frontier is <k), then the algorithm simply requests all the experiments it can.

By construction, one of the experiments selected in Step 3 would have been chosen by the IMGPO algorithm (in the serial optimization setting) given access to the same set of observations. Thus, PROTOCOL has the same convergence guarantees as IMGPO. In particular, both algorithms achieve exponential convergence: $R(x^{+})\in O(\lambda^{N+N_{GP}})$, where λ<1, *N* is proportional to the number of evaluations of the objective (here, the number experiments performed) and *N_GP_* is the number of evaluations of a Gaussian Process model.

At the end of a given pass through the tree, the GP hyperparameters are updated and the algorithm repeats until a prescribed maximum number of evaluations are made.

### 3.1 Descriptive example of algorithm

To illustrate how the algorithm works, we optimize the 1 dimension sinusoidal function $f(x)=\frac{1}{2}(\text{sin}\;13x\;\text{sin}\;27x+1)$ defined over the unit interval (this function is visualized in Supplementary Fig. S1). This function has multiple local maxima over this domain, where the global maximizer is given by x≈0.868. We use a hierarchical tree T to maintain and visualize the division scheme over the input space. Each node corresponds to a subinterval obtained after division of its parent interval node. Each interval is either associated to the ground-truth function evaluated at the interval center, if it has been previously selected, or the UCB evaluated at the center according to the GP model otherwise.

**Fig. 1.**
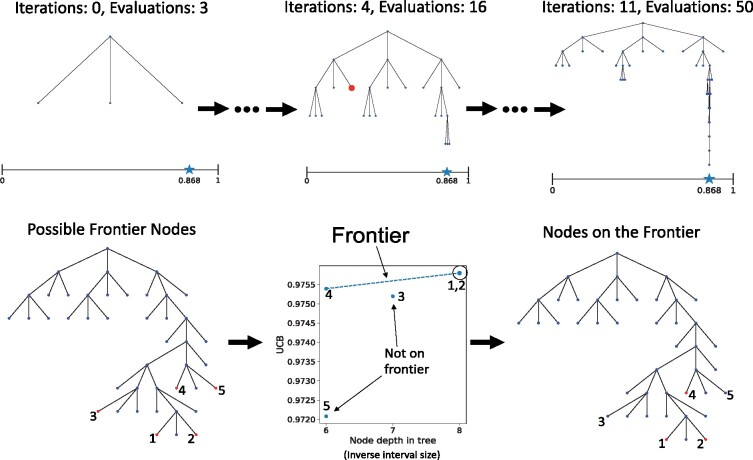
Top row. Shown are hierarchical trees produced by PROTOCOL at three different time points while optimizing a 1D sinusoidal function (see text for explanation). The nodes are fixed along the horizontal axis according the center coordinate of the interval they represent. The function optimizer, x≈0.868, is indicated by the star along the horizontal axis. Bottom row. A visualization of the frontier calculated by PROTOCOL in relation to the hierarchical tree. The enumerated red nodes on the left indicate intervals whose center coordinate are used to calculate the frontier. The central diagram shows the frontier, where intervals 1, 2 and 4 lie on the frontier but intervals 3 and 5 do not. Note that the depth of the tree is inversely proportional to the size of the interval. The red nodes on the right denote those intervals that lie on the frontier, and are those whose center coordinates will be requested for evaluation

Figure 1 (Top) shows the hierarchical tree obtained by the algorithm at three different time points. In these figures, the horizontal axis refers to the input space for the function to be optimized, and the nodes have been fixed along this axis according to each interval’s center coordinate. The ground-truth function optimizer is indicated by the star along the axis at x≈0.868.

The leftmost figure shows $T_{0}$, the tree after initial iteration 0. The root node at depth 0 corresponds to the initial interval center located at the center of the input space (*x *=* *0.5). After evaluating the function at this value, the interval is divided into three subintervals with centers x≈0.167, *x *=* *0.5 and x≈0.833. These intervals correspond to the three nodes at depth 1 in the tree. Since the middle interval has the same center coordinate as its parent, it is associated with its ground-truth value, whereas the other nodes are associated with the UCB of a GP model conditioned on this observed data point. Proceeding through Step 1, the algorithm will iterate over each depth level in the tree and try to identify the best candidate node to divide while keeping track of the current best observed value, $\nu_{\max}$. At depth 0, there is only one node, and that node has already been divided, so no candidate is chosen at this level. By default, $\nu_{\max}$ will be set to the ground-truth value of this interval center. At depth 1, none of the nodes have been divided. The algorithm will identify the interval with the best associated value, and one of the following will occur–

- The selected interval has a ground-truth center value greater than or equal to $\nu_{\max}$. This interval is then added to the candidate list for dividing, and $\nu_{\max}$ is set to this center value.
- The selected interval has a ground-truth center value less than $\nu_{\max}$. In this case, no candidate is added to the list at this depth, and $\nu_{\max}$ is not updated.
- The selected interval has a UCB-based center value, rather than ground-truth (i.e. it has not been previously evaluated). If this happens, a frontier will be calculated and up to *k* many experiments will be requested (at this early stage of the algorithm, there are only two possible intervals available for consideration, so the frontier would not be invoked). Once experiments are completed, the ground-truth observations are then associated with their corresponding interval, and the algorithm will again iterate over the depth 1 nodes until 1 or 2 occur.

If the tree has greater depths, the algorithm moves on to the next depth in the tree, and proceeds until all depths have been visited.

The center of Figure 1 (Top) shows the progression of the algorithm after four more iterations. To illustrate Step 2, suppose that the interval represented by the indicated red node at depth 3 has been selected by Step 1. This step will decide whether or not to keep this interval in the list of intervals to be divided. A tree T′ rooted at the node given by this interval is grown to at most a pre-specified depth by using the same division scheme employed by the algorithm—dividing each interval into three smaller intervals by splitting along the longest dimension. The UCB of each interval center is calculated and associated with its node. The UCB values in T′ are then compared to the center values of nodes at depth greater than three in $T_{4}$. If T′ contains a UCB greater than the center of *any* of these intervals from $T_{4}$, then the interval is kept in the list. Otherwise, it is removed.

At the conclusion of Step 2, all intervals that remain selected are then divided. Upon division of a given interval, two of the newly created intervals will have unevaluated centers, while the middle interval will inherit the ground-truth value of its parent. The algorithm then decides whether or not to evaluate each of the two unevaluated centers by comparing the UCB evaluated at the center to $\nu_{\max}$. If the UCB is less than $\nu_{\max}$, then the UCB is used as the interval center. Otherwise, the ground-truth value must be obtained. This is where most calls to calculate the frontier occur.

Figure 1 (Bottom) shows an example frontier. For visual clarity, the horizontal axis has not been directly fixed according to the interval coordinates. In the leftmost figure, nodes 1 and 2 are the two nodes created upon division, and nodes 3, 4 and 5 are the others that are considered for the frontier (i.e. they are the remaining leaf nodes whose centers have not been previously evaluated). The middle figure visualizes the frontier, where nodes 1, 2 and 4 are selected. Nodes 1 and 2 are selected because they were the two new nodes created by division (they happen to fall on the frontier, but would have been evaluated regardless). Node 3 is not selected because it does not fall on the upper convex hull, and node 5 is not selected because it is dominated by node 4, which has a greater UCB and is at the same depth in T. The selected interval centers are then evaluated, and their ground-truth values are associated with each corresponding node.

The algorithm repeats until a prescribed number of evaluations are made. The rightmost figure in Figure 1 (Top) shows the final tree $T_{11}$ obtained after 50 function evaluations. Notice how the width of the tree indicates that the algorithm was able to explore the initially unknown search space, while the depth of the tree is largely concentrated near the optimizer x≈0.868. This shows that the GP model was able to quickly guide the division toward this global optimal solution.

## 4 Experiments and results

### 4.1 Simulated Cloud Lab with real data—MALDI-ToF MS protocol optimization

Matrix-assisted laser desorption/ionization time of flight (MALDI-ToF) mass spectrometry is a laboratory method used to characterize the contents of a sample, and is used across many scientific domains (Jang and Kim, 2018; Spraggins *et al.*, 2016). The end result of this experiment is a spectrum that is used to identify the components within the sample. Significantly, a variety of instrument settings must be specified, and these adjustable parameters affect the quality of the resulting data. Typical user-specified parameters include: (i) the accelerating voltage, (ii) the grid voltage, (iii) the pulse delay and (iv) the number of laser shots per spectrum.

When performing MALDI-ToF spectrometry, it is common to perform a brute-force parameter sweep in order to identify the configuration that produces the highest quality spectrum. We were provided access to the data produced by two MALDI-ToF parameter sweeps for two separate samples. The biological context for these experiments was a study for the use of enzyme-polymer conjugation for chymotrypsin enzyme replacement therapy (Cummings *et al.*, 2017; Kaupbayeva and Russell, 2020). The two samples used in these experiments included one with native chymotrypsin (CT), and the other with a chymotrypsin-polymer conjugate (CT-polymer). The goal of the parameter sweeps was to identify configurations that produce easily identifiable signals from each sample. Since the CT-polymer conjugate is a more complex sample, it should be expected to be harder to obtain such an identifiable signal.

Each dataset consists of 120 MALDI-ToF spectra produced via a manual, brute-force grid search over the four user-specified parameters named previously. We ran PROTOCOL in a simulated cloud lab environment to demonstrate that the algorithm can identify the optimal parameter configuration in many fewer experiments. By simulated, we mean that the results of each experiment submitted to the job queue is simply fetched from the given datasets.

Our experiments considered several different definitions of spectral quality. We used the MATLAB Bioinformatics Toolbox to calculate peak height (intensity), peak width and signal-to-noise ratio (SNR) of each spectrum. In general, a strong signal will include a large peak, narrow width and small SNR. Additionally, as a means of combining these properties into objective measures that quantify multiple properties at the same time, we also used two linear combinations of these endpoints:

(2) $$\text{Combo}1=\text{Peak}\;\text{Height}+\frac{1}{\text{Peak}\;\text{Width}}$$

 

(3) Combo2=SNR+Combo1

The peak height, peak width and SNR measurements were first scaled to the unit interval to ensure that different underlying distributions of each endpoint did not skew the objective.

For both datasets, we ran *in silico* experiments using PROTOCOL that optimized for each of these endpoints. For each endpoint, this entails sequentially observing ground-truth values for experimental configurations according to the scheme outlined in Section 3. Each of the four input parameters are scaled to the unit interval, which transforms the input space onto the unit hypercube, as is done with IMGPO. An important difference between this setting and the example using the 1D sinusoidal function is that we choose experiments from one of 120 possible configurations. To do this, we use the division procedure outlined previously, but instead of evaluating the center coordinate of an interval, we calculate the Euclidean distance between the center coordinate and each transformed parameterization, and assign the closest parameter setting to the interval, where each parameterization is only allowed to be used once. In general, PROTOCOL can also handle any non-continuous variables in this way, so long as the variables are numerically encoded.

In our experiments, we used a GP with Matérn kernel with ν=5/2, and initialized the hyperparameters *σ*  =  1 and *l *=* *0.25. These hyperparameters are optimized by maximizing the log marginal likelihood at the end of each iteration. Given appropriate priors over the hyperparameters, they may be sampled from this distribution, though we omit this procedure from our experiments. Other IMGPO-specific hyperparameters were set to their default settings. We set the level of parallelization to four, meaning that PROTOCOL could request as many as four experimental conditions to observe at a time. In each experiment, we allowed the algorithm to select a total of 25 observations. This corresponds to having only run 25 experiments in the simulated lab, as opposed to the complete set of 120, as was done in reality. As points for comparison in a parallel setting, we also performed batch-mode GP optimization on the same data using standard acquisition procedures, including Thompson sampling (TS), UCB, EI and PI. Each of these methods choose four configurations to observe according to the acquisition procedure, update their GP model (including updating GP hyperparameters), then choose again using the updated model. The initial setting of the GP in each of these were the same as with PROTOCOL, and fully exhausting the 25 experiment request budget was used as the stopping criterion. We repeated each 100 times with different randomly selected training sets of size equal to the level of parallelization (in this case, 4). This imitates the most efficient way one could initiate each of these procedures in a real cloud lab setting.

#### MALDI-ToF simulated cloud lab results

In both MALDI-ToF datasets, we find that PROTOCOL is able to find the optimal parameter configurations in most situations. Tables 1 and 2 show the probability that PROTOCOL and other GP optimization algorithms identify the optimal parameter configuration for each endpoint. As PROTOCOL is a deterministic algorithm, it can only take values 100% or 0%. The other probabilities are calculated as the number of times each found the optimal setting out of the 100 repeated experiments. There was only one case where PROTOCOL did not find the optimal configuration (SNR, CT dataset), whereas the other GP-based algorithms have low success rates (mean =34%; median =36%; SD =13.4%; max =69%). Notably, PROTOCOL was able to identify the optimal configuration for each endpoint with the more difficult CT-polymer dataset. Comparably, the performance of the other GP optimization regimes tended to decrease with the CT-polymer dataset compared to just native CT.

**Table 1.** The probability that each algorithm identifies the optimal experimental parameterization with the Native CT data for each endpoint

Algorithm	Height	Width	SNR	Combo1	Combo2
PROTOCOL	100%	100%	0%	100%	100%
TS	43%	9%	40%	35%	38%
EI	35%	11%	69%	40%	38%
PI	36%	40%	63%	40%	38%
UCB	42%	9%	42%	36%	29%
Random	25%	19%	24%	23%	18%

**Table 2.** The probability that each algorithm identifies the optimal experimental parameterization with the CT-polymer conjugate data for each endpoint

Algorithm	Height	Width	SNR	Combo1	Combo2
PROTOCOL	100%	100%	100%	100%	100%
TS	42%	50%	41%	21%	31%
EI	38%	54%	53%	25%	26%
PI	39%	56%	52%	25%	26%
UCB	43%	53%	37%	28%	28%
Random	21%	31%	19%	22%	18%

**Fig. 2.**
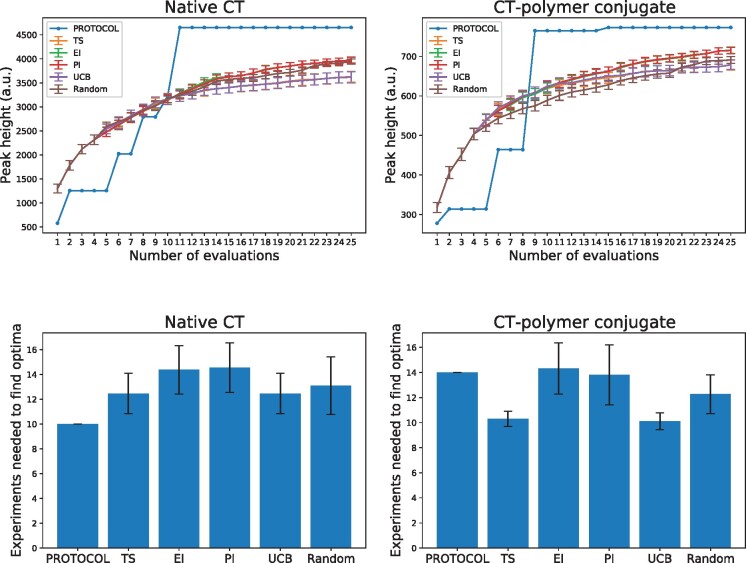
Top row. The ground truth peak height of observed MALDI-ToF experimental configurations is shown as a function of the number of total evaluations. The error bars in the non-PROTOCOL curves denote a mean ± 1 SEM calculated over 100 trials initialized with different randomly chosen training sets of size 4 (which is equal to the allowed level of parallelization). Bottom row. Again with the peak height endpoint, these show the number of evaluations each algorithm requested before identifying the optimal configuration. For the non-PROTOCOL algorithms, only the subset of the 100 trials that actually identified the optimal configuration are used. Error bars denote ± 1 SEM over this subset of trials

To succinctly describe the selection behaviors of each algorithm, we focus our next analyses on the peak height endpoint, though similar summaries could be made for others. Figure 2 (Top) shows the average progress of each algorithm in identifying the optimal experimental parameterization. That is, it shows the best observed value as a function of number of experiments requested and conducted. Experiments with both native CT and CT-polymer conjugates yield similar patterns. Initially, PROTOCOL lags behind the other GP optimization algorithms, but then quickly rises to the top and identifies better protocols (in the case of peak height, the *best* available protocol). We emphasize that the apparent success of the random procedure is due to the relatively few experiments available (120) to be chosen from 25 times without replacement. Tables 1 and 2 show that the random procedure finds the true optimum much less frequently than the other conventional BO approaches, as expected. To demonstrate these results generalize beyond these specific datasets, we additionally ran experiments optimizing general-purpose optimization functions, and obtained similar results (Supplementary Fig. S2).

As already described, the comparison GP algorithms require an initial training batch, and are only able to identify the optimal parameterization a fraction of the time depending on this initial training data. Figure 2 (Bottom) focuses on the subset of trials that were able to correctly identify the optimal protocol parameterization. Over these trials, it shows how many experiments were requested on average before the optimal configuration was chosen. The native CT data PROTOCOL needed only 10 experiments, while the other methods required on average 12–14 experiments. This suggests that even when the other GP algorithms are able to identify the optimal experimental parameterization, PROTOCOL is capable of identifying the optimal solution more quickly.

**Fig. 3.**
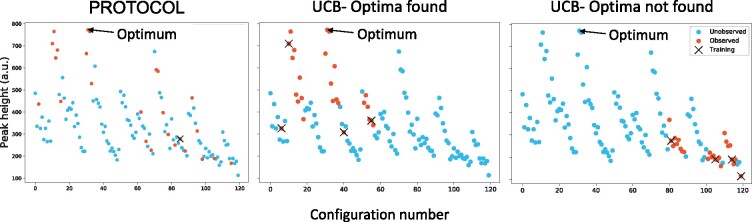
CT-polymer conjugate ground-truth peak heights for MALDI-ToF parameterizations selected by PROTOCOL and the GP-UCB algorithm. Two cases are shown for the GP-UCB algorithm—one where the algorithm identified the configuration that led to the maximum peak height, and one that did not. For each, the initial evaluation points are indicated by an ‘x’. Whereas the initial point evaluated by PROTOCOL is a consequence of the algorithm (the central point of the input space), GP-UCB depends on an initial training set. The ability of GP-UCB to identify the optimal configuration is influenced by this initial set

With the CT-polymer conjugate data, TS and UCB seem to identify the optimal configuration in fewer experiments compared to all other methods (although, as previously mentioned and shown in Table 2, they find such optimal configurations less than 57% of the time). To investigate this behavior further, Figure 3 visualizes choices made by PROTOCOL compared to two trials that used GP-UCB—one that identified the optimal protocol and one that did not (results with TS are similar). In the figure, the configurations are enumerated along the horizontal axis, with the peak height of the spectra produced by the given experiment along the vertical axis. In general, configuration numbers closer to each other correspond to experimental configurations that are more similar to each other.

PROTOCOL’s initial experiment corresponds to an experiment that is far from optimal. Still, the algorithm is able to quickly survey the input space, and converge on experimental configurations that yield large peak heights. With the UCB algorithm, the behavior is largely dependent on the initial training set that was randomly chosen. When there are training instances that are similar to the optimal configuration, the algorithm successfully identifies the optima, but is prone to converging on local optima more similar to the training data otherwise. This suggests that PROTOCOL is better at escaping local optima than the comparison algorithms.

In Supplementary Figure S3, we show results from experiments where we varied the level of parallelization from *k *=* *1 to *k *=* *10, using both MALDI-ToF datasets, as well as with three commonly used optimization functions. For each data and choice of *k*, we see that PROTOCOL performs the best.

### 4.2 Real-world Cloud Lab—HPLC protocol optimization

High performance liquid chromatography (HPLC) is an analytical chemistry technique used to separate and quantify components of complex mixtures. The method uses pressurized liquid solvent to force a sample through a column containing specialized solid adsorbent material. This material interacts with each component of the sample differently, causing each to travel through the column at different rates, thus separating the mixture. A detector measures the absorbance of each sample as they elute at different times. Chromatograms are generated from these measurements, which allows for identification of each component (Thammana, 2016).

We used PROTOCOL to optimize an HPLC experimental design in a real cloud lab setting using Emerald Cloud Labs (ECL). This means that whenever PROTOCOL requested an experimental configuration to observe, we remotely executed an actual experiment to be performed at ECL’s laboratory in South San Francisco, CA. Specifically, the HPLC experiment involved the separation of a three component mixture of the organic compounds phenol, toluene and 2,5-xylenol in a water/methanol solvent. The accuracy of the separation and quantification steps are sensitive to multiple parameters. In each experiment, we chose a setting for each of the following parameters:

- **Flow rate—**The speed of the fluid through the HPLC pump. Selected from the range 0.2–2 mL/min.
- **Injection volume—**The physical quantity of sample loaded into the flow path for measurement. Selected from the range 1–50 μL.
- **Column temperature—**The temperature of the HPLC column. Selected from the range 25–45°C.
- **Absorbance wavelength—**The wavelength used by the detector to identify samples flowed through the column. Selected from 260–285 nm.
- **Solvent ratio—**The proportion of methanol to water in the solvent. Selected from 65:35, 70:30, 75:25 and 80:20.
- **Gradient—**The proportion of solvent: sample flowed through the column over time. Selected from a non-linear (the default ECL setting), constant, quick linear, linear and slow linear setting.

Unlike the MALDI-ToF experiments where we wanted to optimize the signal from a single sample with a single component, here we want to simultaneously optimize for the sample (by adjusting solvent ratios) *and* the instrument settings that best resolve the three compounds mixed within a sample. To do this, we used the resolution of the chromatogram (*R_S_*) as our objective function. This is a commonly used metric to describe HPLC spectra, and is given by:

(4) $$\begin{array}{c} R_{S}=\sum_{i=1}^{n-1}\frac{c(t_{i+1}-t_{i})}{w_{i}+w_{i+1}} \end{array}$$

where *n* is the number of peaks, *t_i_* refers to the time component *i* elutes from the HPLC column, *w_i_* refers to the half-height width of component *i*, and *c* is a constant that arises from assuming each peak takes the shape of a Gaussian. Essentially, this objective quantifies how clearly distinguished all adjacent peaks are from each other. The larger the value, the more clear the separation. We used ECL’s built in software in order to pick peaks from the chromatogram.

We had access to three threads on ECL, meaning we could run up to three experiments at a time. We thus allowed PROTOCOL to select up to this many experiments per request. Since each possible parameter configuration was either chosen from a finite list (gradient and solvent ratio) or subject to a finite level of precision when measuring (flow rate, injection volume, column temperature and absorbance wavelength), we used the same strategy as with the MALDI-ToF experiments when assigning a parameter configuration to an interval within PROTOCOL’s hierarchical tree. The input space was similarly scaled to the unit hypercube, and we used the same GP and IMGPO specific hyperparameters detailed in Section 4.1.

Since we were running real experiments with a limited number of threads on ECL, we did not have the time or resources to conduct experiments according to alternative acquisition functions (TS, EI, etc.) as with the MALDI-ToF simulated cloud lab data. Thus, we also used Latin Hypercube Sampling (LHS) as a baseline to select experiments to conduct on ECL. Unlike with PROTOCOL and other conventional BO strategies, this is not a sequential process, but rather a form of randomly sampling a prescribed number of experiments to conduct. This allowed us to submit all the experiments at once, and allow them to complete according to available resources without the need for further intervention. We then compared the results of the LHS versus PROTOCOL in a separate set of experiments. In total, we executed 18 experiments selected by PROTOCOL, and (separately) 18 experiments selected via LHS on ECL. Given our available resources, this was the number of experiments we estimated could be run in 2 month’s time (1 month for each approach).

#### HPLC real cloud lab results

PROTOCOL and LHS are both able to identify HPLC configurations that yield high resolution. Table 3 shows the top three scoring resolutions obtained from configurations selected by both methods. While PROTOCOL’s top scoring configuration yielded a resolution of 17.4, strikingly, LHS selected a configuration that obtained a resolution of 44.1. Since LHS is ultimately a random sampling procedure, it was unexpected that it was able to identify such a highly resolved spectrum. This led us to investigate precisely how unlikely this finding was.

**Table 3.** The top 3 HPLC spectra resolutions according to configurations chosen by PROTOCOL and LHS

Algorithm	Best	2nd best	3rd best	Average
PROTOCOL	17.4	16.4	8.5	14.1
LHS	44.1	11.5	2.9	19.5
Sim.-LHS	15.8 ± 2.6	12.2 ± 1.5	7.4 ± 1.5	11.8

*Note*: Sim.-LHS refers to a simulated LHS method (see text for details), where mean ± 1 standard deviation are shown over 500 runs. ‘Best’ refers to the greatest observed resolution.

We accomplished this through simulation. We trained a random forest regression model with the 36 experiments obtained from PROTOCOL and LHS, using the measured resolution as the label. We then repeated the LHS sampling procedure used to generate experiments 500 times. This allowed us to predict the resolution of these samples using the random forest regression model. The result of these 500 LHS simulations are shown by Sim.-LHS in Table 3 as a mean ± 1 standard deviation.

We find that the Sim.-LHS results do not yield resolutions anywhere near as large as the 44.1 found by the experiments on ECL. Rather, the best prediction on average had a resolution of 15.8. For reference, PROTOCOL’s best is about one standard deviation larger than this value. Furthermore, the maximum predicted resolution over all 500 simulations was only 26.9, giving a 39% difference in the maximum resolution observed in the ECL experiments. This is evidence that the configuration that yielded a 44.1 was highly unlikely to have been chosen.

The best experimental configurations selected by PROTOCOL and LHS were quite different. PROTOCOL’s best configuration is given by Flow rate = 1 mL/min, Injection vol. = 38.3 μL, Column temp. = 35°C, Absorbance wavelength = 273 nm, solvent ratio = 75:25 and Gradient = quick linear. LHS’ best is given by Flow rate = 0.8 mL/min, Injection vol. = 6.1 μL, Column temp. = 26.7°C, Absorbance wavelength = 284 nm, solvent ratio = 65:35 and Gradient = non-linear. PROTOCOL not finding a similar configuration suggests that, within this search space, more than 18 experiments were needed. We emphasize that the choice to perform 18 experiments was a product of time and resource constraints.

**Fig. 4.**
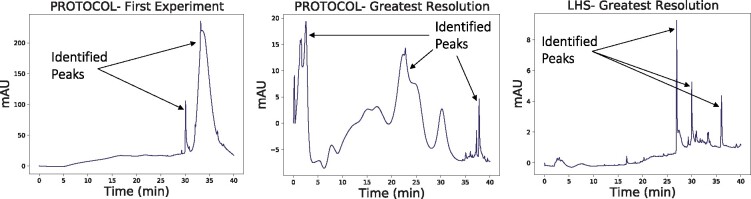
Chromatograms corresponding to the first experimental configuration chosen by PROTOCOL (left) as well as the experimental configuration that yielded the greatest resolution chosen by PROTOCOL (middle) and LHS (right)

Figure 4 shows peaks that correspond to experiments selected by PROTOCOL and the outlier LHS result. The panel on the left is typical of the chromatogram one obtains using a configuration chosen at random (including those *typically* chosen via LHS). The panel on the right is an example of a reasonably high quality chromatogram, albeit one that is unlikely to have been observed when using LHS, as previously argued. The middle panel is the best one found by PROTOCOL given a budget of 18 experiments. It is clearly an improvement over the left panel. In particular, the built-in automatic peak picking and resolution-calculating software only identified two peaks in the right panel, but identifies three peaks in the middle panel (the correct number). Still, the middle panel is far from optimal. We hypothesize that given a larger experiment budget, PROTOCOL would have continued to find better configurations. Moreover, as shown in the bottom row of Table 3, the typical best resolution obtained via LHS is actually lower than that of the middle panel. That is, the middle panel is probably representative of what one might obtain via a LHS with a budget of 18 experiments over this search space.

As a final note, we emphasize the advantage PROTOCOL’s use of parallelism has over purely serial procedures (such as IMGPO). While we only ran a modest number of experiments selected by PROTOCOL (18), it still took 26 days to complete. Had we had run each experiment sequentially one after another, we estimate from queue times and experiment execution times that it would have taken *at least* 41 days, meaning we saved greater than 15 days worth of work.

## 5 R Shiny application

**Fig. 5.**
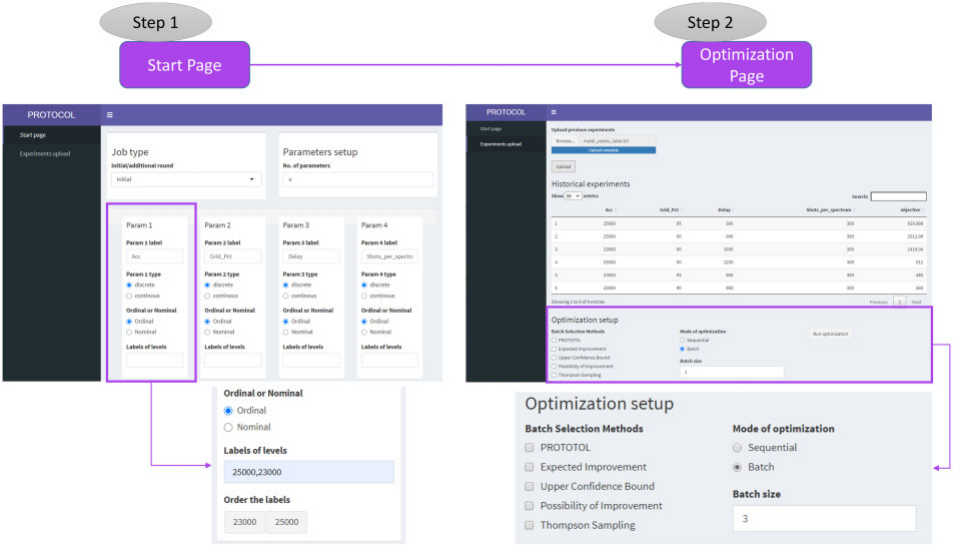
Left. The Shiny app start page, where the user can initialize an optimization problem by defining the parameters to optimize over. Right. The Shiny app data upload page, where the user can upload previously evaluated data and select the optimization algorithm to use

We have implemented PROTOCOL as both a Python library, and as part of a stand-alone application written in R Shiny (Chang *et al.*, 2020) (see Availability). The Shiny app lets the user initialize and run optimization jobs using PROTOCOL or several conventional BO methods (TS, EI, PI, UCB). The user defines the optimization problem via a start page by specifying the names and types of the parameters to optimize over (by hand, or by loading a configuration file Fig. 5 (Left). After confirming all parameters, the users can upload historical parameters combinations with their observed objective values (if available). The users then selects the optimization method (e.g. PROTOCOL) and the degree of parallelism (*k*). If desired, the user can select more than one optimization method to consider different selections Fig. 5 (Right). The application computes and displays the next parameter combination(s) to run. Any existing data and the suggested combinations can be saved to a file, in order to save state, as the user waits for the experiments to run. When the results of the experiments are known, the user updates the file and then loads it into the application, which then suggests the next experiment(s) to run, and so forth.

## 6 Discussion and conclusion

Cloud-based laboratories present an emerging and exciting new model for conducting scientific experiments. While they can provide access to sophisticated equipment and the ability to run experiments in parallel, performing experimental optimizations in a way that fully utilize these capabilities is an unexplored area. To this end, we have developed PROTOCOL, an algorithm that performs closed-loop optimization of experimental protocols within this setting. Built on the framework of recent bound-based optimization methods that come with convergence guarantees, we believe PROTOCOL to be the first such method that explores optimization of experimental designs within this environment.

In our MALDI-ToF experiments, we compared PROTOCOL to conventional BO approaches. We found that PROTOCOL more reliably identified the optimal configuration across five different endpoints and with two different samples. The ability of the conventional approaches to select desirable parameterizations is highly dependent on the initial data used to train the models. While there are ways to promote exploration of the search space with such GP-based approaches, this itself entails an auxiliary optimization routine, which could be computationally prohibitive and/or prone to over-fit limited data. We found that PROTOCOL’s DIRECT-like division scheme over the input space was able to combat these issues.

The deterministic nature of the dividing scheme ensures that the underlying GP model is exposed to instances that are representative of a wide range of the input space, so it naturally promotes exploration. Additionally, whereas the conventional approaches select parameterizations primarily based on what the model believes to be best at any given time, PROTOCOL’s strategy is to present the model with parameterizations selected according to the division scheme, and requesting experiments the model is sufficiently uncertain about. This ensures that selections made by PROTOCOL are not as exclusively influenced by what the model has been exposed to previously, which leads to a regularization-like behavior. Another area for future work is to integrate the results of technical and/or biological replicates into the optimization logic.

While our work with PROTOCOL is a promising start toward experimental optimization in real Cloud Lab settings, there is still much room for improvement. In our HPLC experiments using ECL, we noted that PROTOCOL likely needed more than 18 selections to identify an experiment as well resolved as the anomalous LHS finding. Given that our search space over HPLC parameters was orders of magnitude larger than that of the MALDI-ToF parameters, it is not altogether surprising that a larger number of experiments could be necessary.

There are a number of algorithmic improvements worth pursuing. This includes identifying better strategies to initialize PROTOCOL’s hierarchical tree. At present, the algorithm always starts by selecting the center of the hyperrectangle, no matter how much prior data are available. An alternative approach might initialize a tree that is already grown to some depth, and using some subset of leaf nodes in this tree as a starting point. The use of the frontier would provide a natural way to still select from nodes that were created in the tree’s initiation. Since this tree could provide a larger initial pool of experiments to choose from, it could help provide a better initial search in larger dimensional settings.

In this work, we have shown how to adapt a principled bound-based BO routine to work in a parallel setting. We further demonstrate that such an approach is capable of being executed in a real cloud lab environment, and show that it performs favorably relative to conventional BO routines on real-world data. While we chose to adapt PROTOCOL from IMGPO due to its theoretical guarantees, there are of course other sophisticated BO algorithms that could be similarly adapted to work in our cloud-based setting (e.g. Tarun *et al.*, 2016). We leave formal comparisons between the performance of such alternative approaches in the cloud lab to future work.

One final point is that PROTOCOL is broadly more applicable than the experimental design use case we have presented here. Since it shares similarities with other bound-based optimization algorithms (most directly IMGPO), it could be used to optimize most any black-box function. It would be interesting to apply PROTOCOL to tackle such problems in parallel, such as hyperparameter optimization for deep models, especially those applied to biological settings.

## Supplementary Material

btab291_Supplementary_Data
