# Supplementary material for: Asynchronous parallel Bayesian optimization for AI-driven cloud laboratories
Source: Bioinformatics. 2021 Jul 12;37(Suppl 1):i451–9. doi: 10.1093/bioinformatics/btab291 (PMC8275326; doi:10.1093/bioinformatics/btab291)
Supplement: btab291_Supplementary_Data [file btab291_supplementary_data.zip › btab291-suppl_data/Frisby.78.sup.3.pdf]

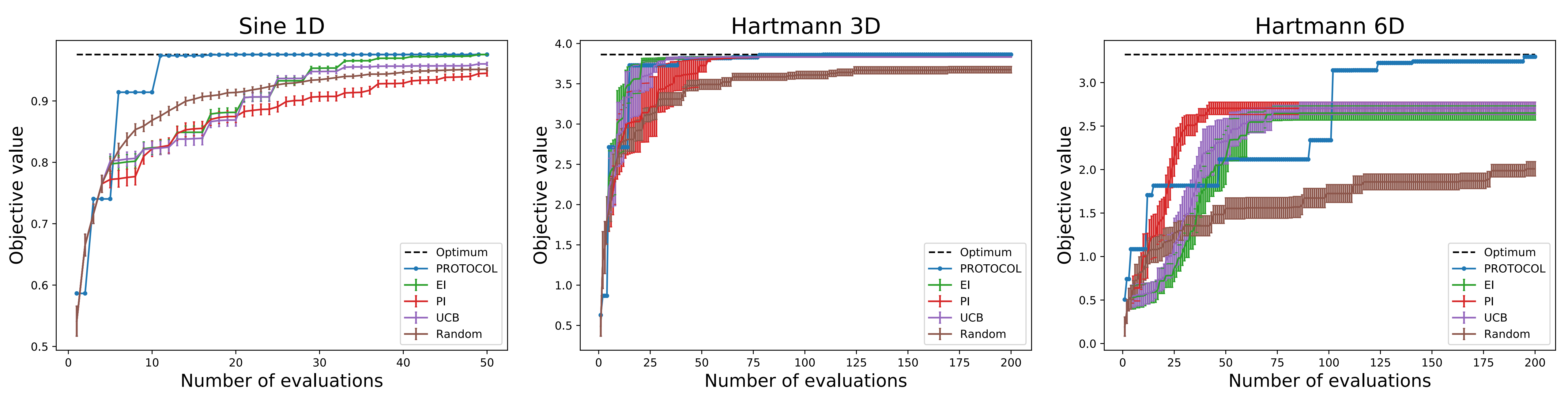
Sine 1D
Hartmann 3D
Hartmann 6D
Objective value
Number of evaluations
Optimum
PROTOCOL
EI
PI
UCB
Random

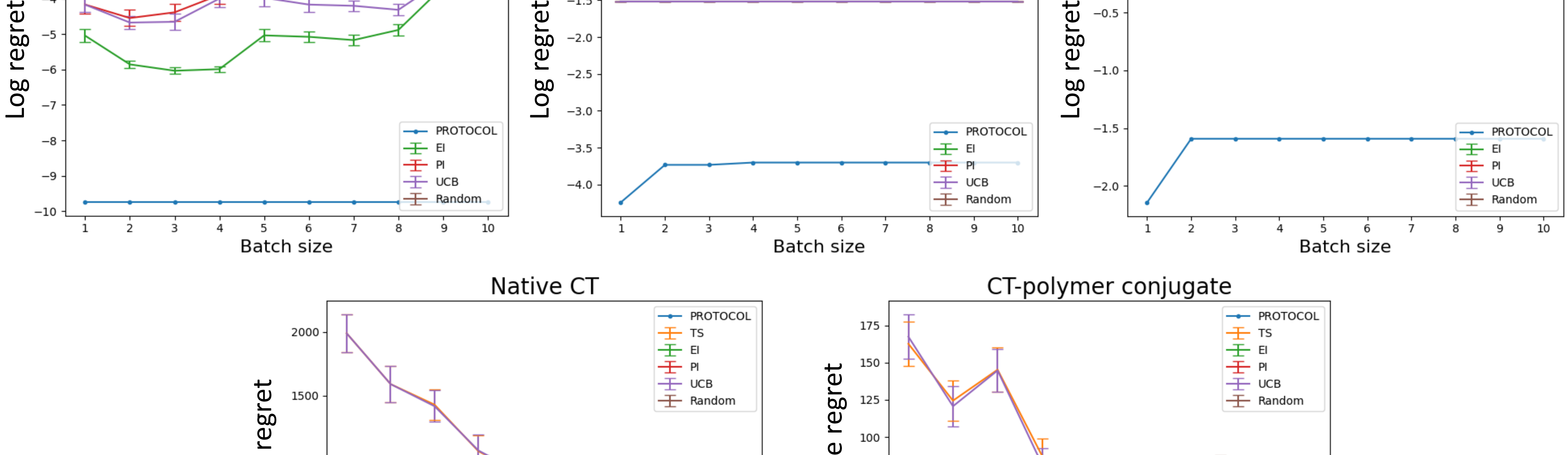
Log regret
Batch size
PROTOCOL
EI
PI
UCB
Random
Log regret
Batch size
PROTOCOL
EI
PI
UCB
Random
Log regret
Batch size
PROTOCOL
EI
PI
UCB
Random
Native CT
regret
PROTOCOL
TS
EI
PI
UCB
Random
CT-polymer conjugate
PROTOCOL
TS
EI
PI
UCB
Random

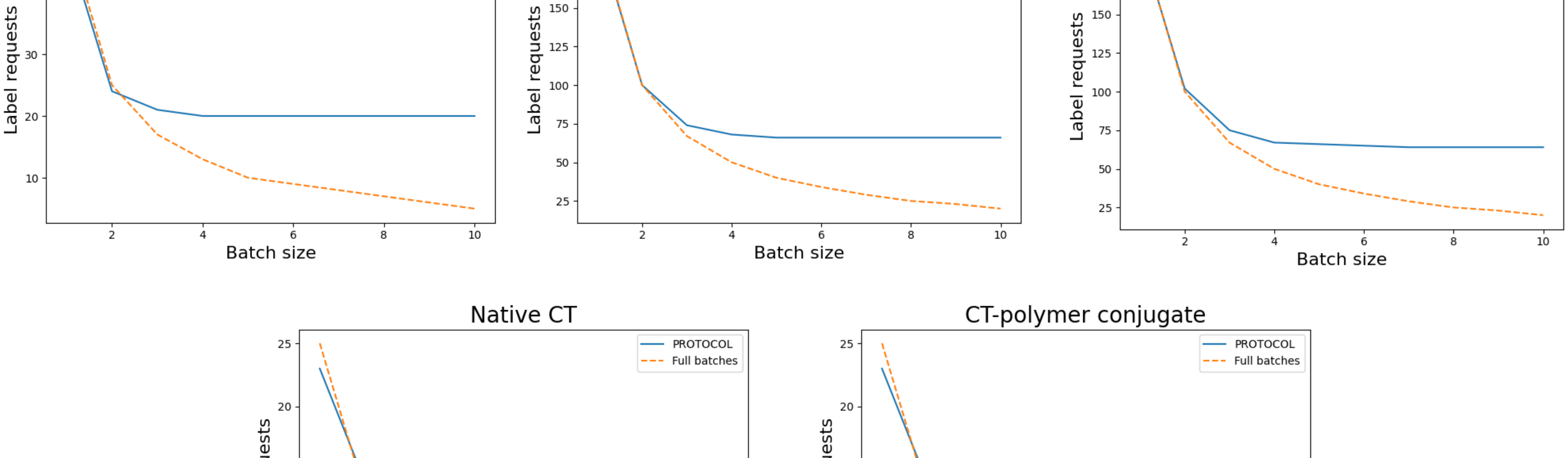

Label requests
Batch size
Native CT
CT-polymer conjugate
PROTOCOL
Full batches

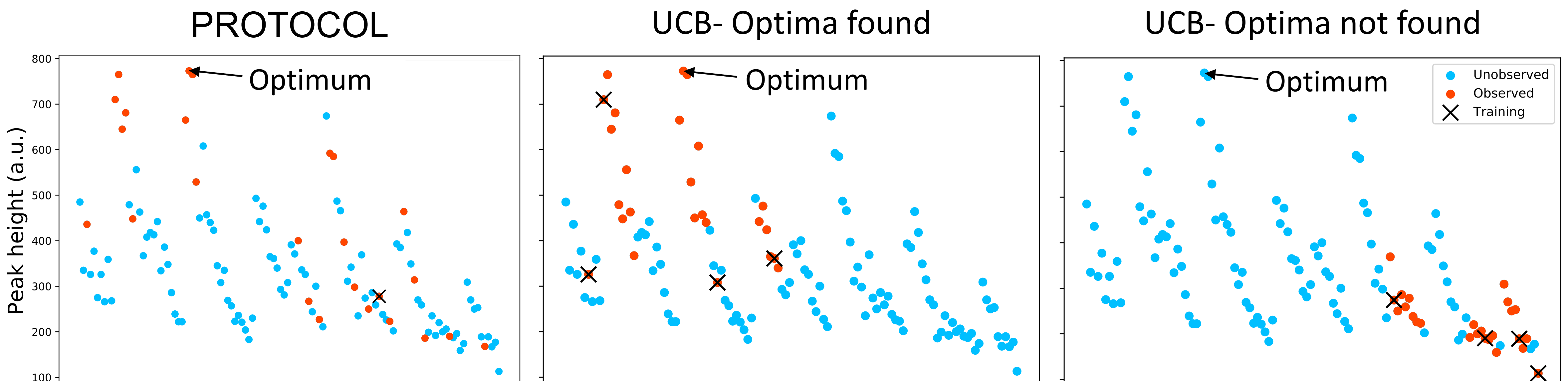
PROTOCOL
UCB- Optima found
UCB- Optima not found
Optimum
Optimum
Optimum
Peak height (a.u.)
800
700
600
500
400
300
200
100
Unobserved
Observed
Training
