# Supplementary material for: Asynchronous parallel Bayesian optimization for AI-driven cloud laboratories
Source: Bioinformatics. 2021 Jul 12;37(Suppl 1):i451–9. doi: 10.1093/bioinformatics/btab291 (PMC8275326; doi:10.1093/bioinformatics/btab291)
Supplement: btab291_Supplementary_Data [file btab291_supplementary_data.zip › btab291-suppl_data/Frisby.78.sup.4.pdf]

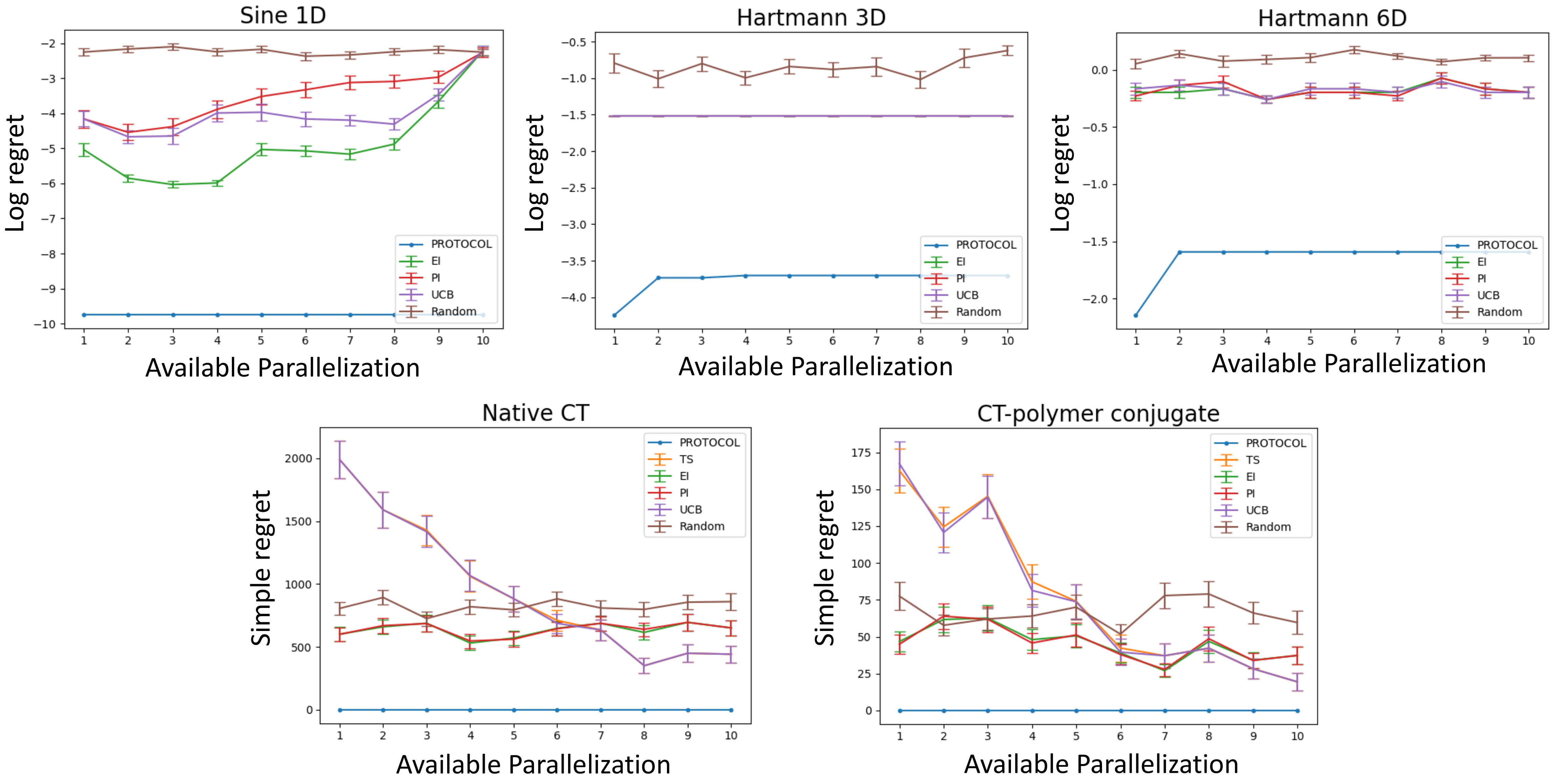
Sine 1D
Hartmann 3D
Hartmann 6D
Native CT
CT-polymer conjugate
Log regret
Simple regret
Available Parallelization
PROTOCOL
TS
EI
PI
UCB
Random

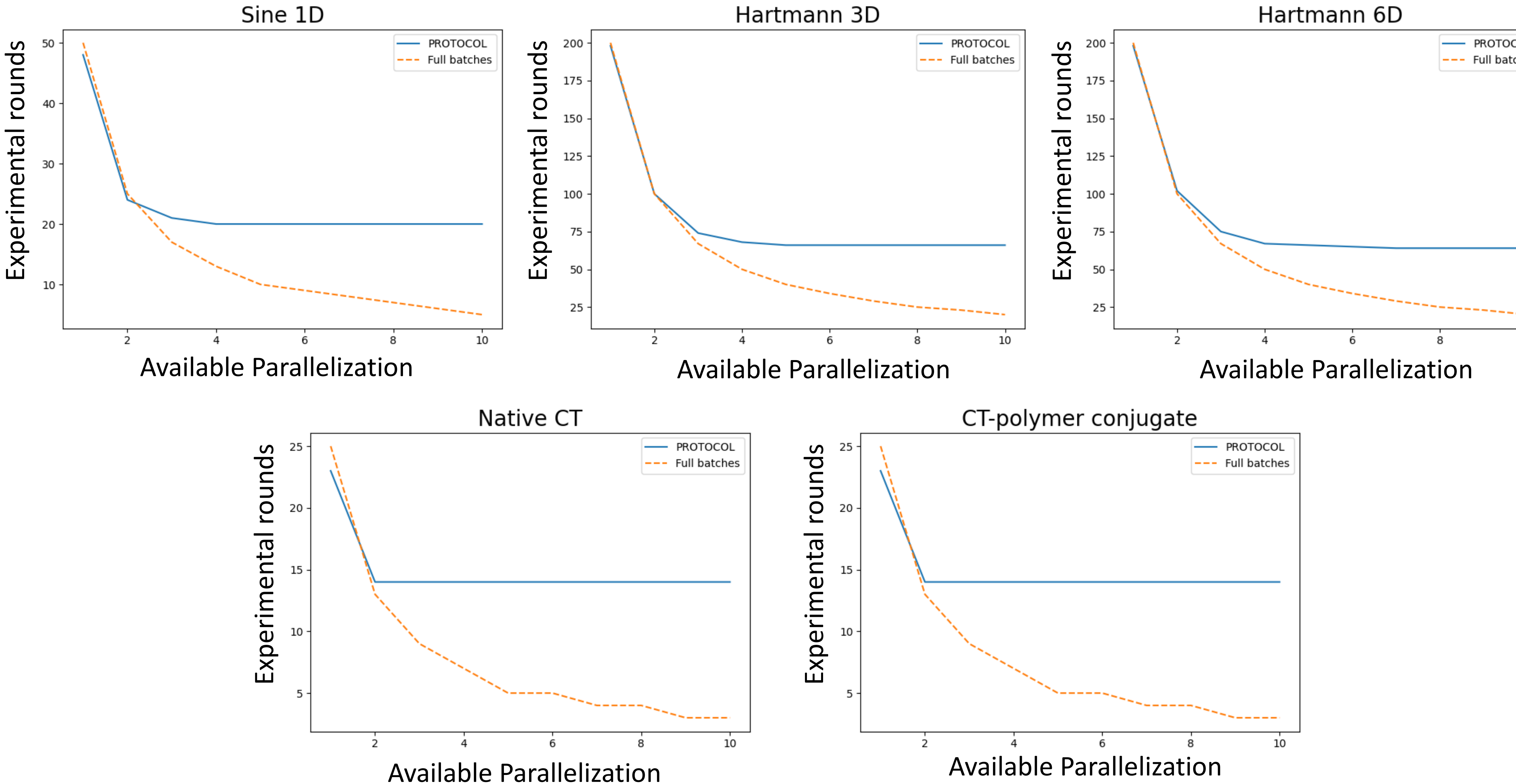

Sine 1D
Experimental rounds
Available Parallelization
PROTOCOL
Full batches
50
40
30
20
10
2
4
6
8
10
Hartmann 3D
Experimental rounds
Available Parallelization
PROTOCOL
Full batches
200
175
150
125
100
75
50
25
2
4
6
8
10
Hartmann 6D
Experimental rounds
Available Parallelization
200
175
150
125
100
75
50
25
2
4
6
8
Native CT
Experimental rounds
Available Parallelization
PROTOCOL
Full batches
25
20
15
10
5
2
4
6
8
10
CT-polymer conjugate
Experimental rounds
Available Parallelization
PROTOCOL
Full batches
25
20
15
10
5
2
4
6
8
10

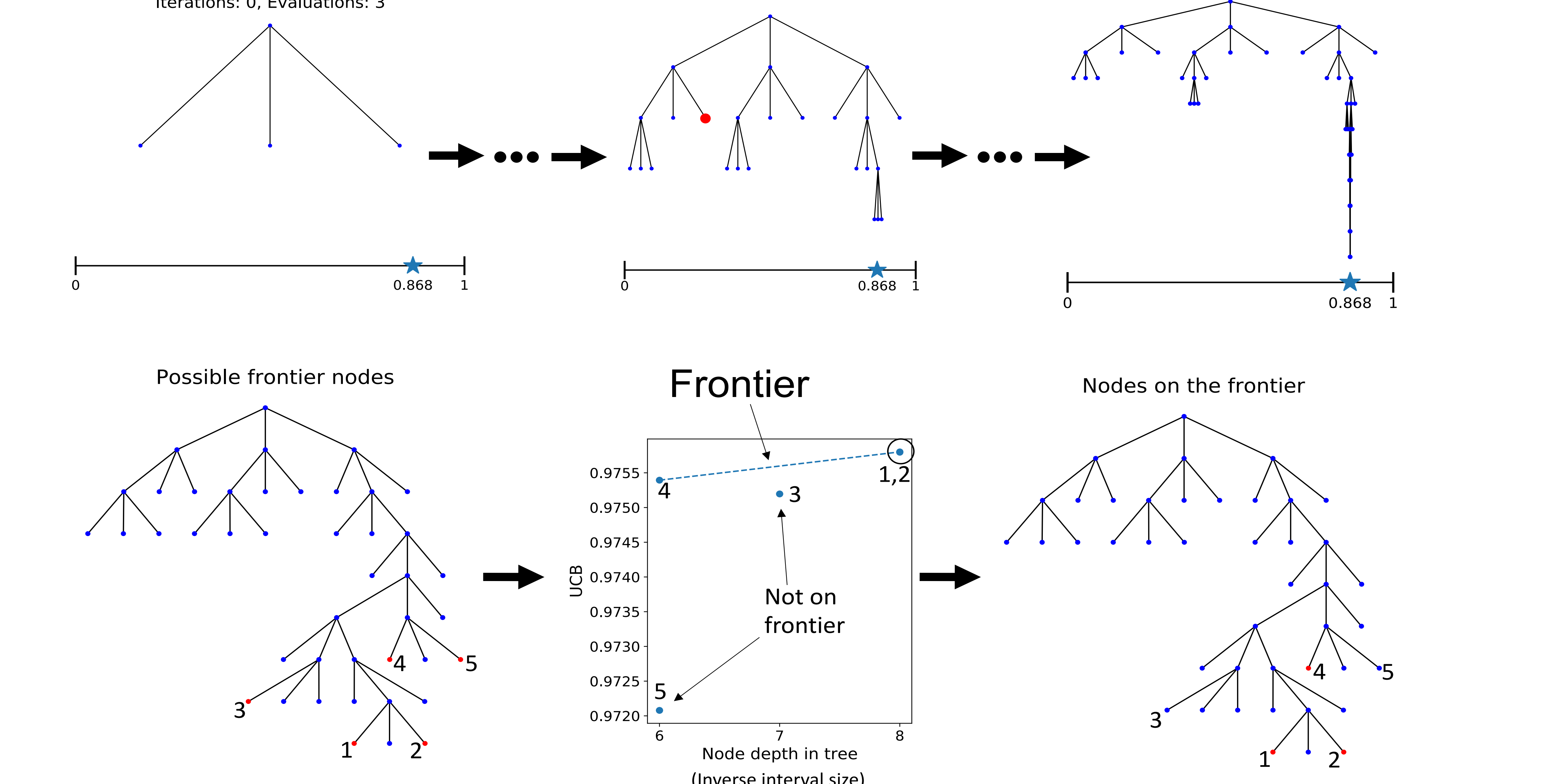
Iterations: 0, Evaluations: 3
0
0.868
1
0
0.868
1
0
0.868
1
Possible frontier nodes
3
1
2
4
5
Frontier
UCB
0.9755
0.9750
0.9745
0.9740
0.9735
0.9730
0.9725
0.9720
4
1,2
3
Not on
frontier
5
6
7
8
Node depth in tree
(Inverse interval size)
Nodes on the frontier
4
5
3
1
2

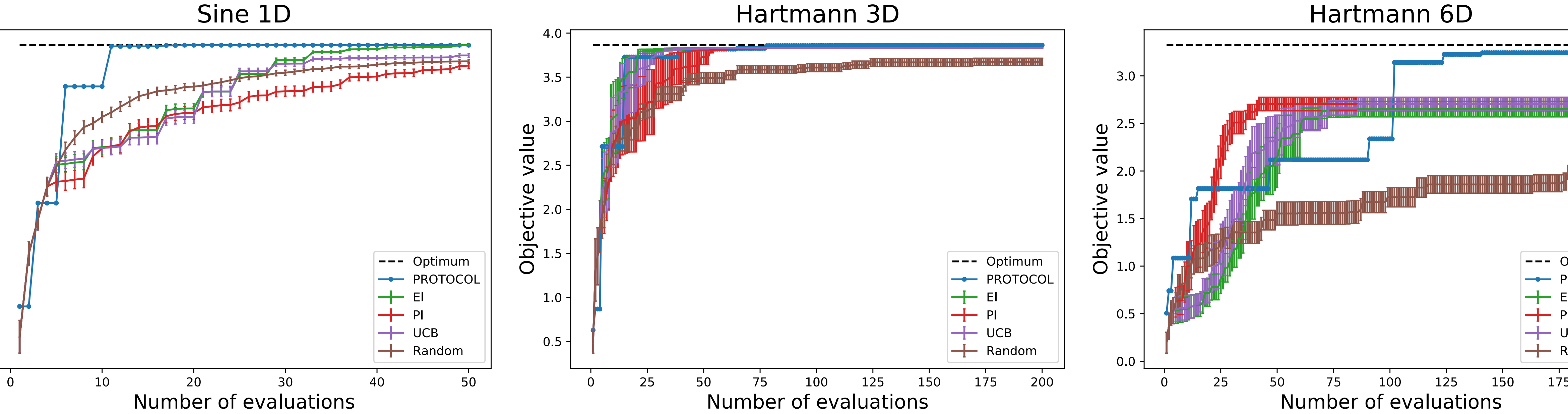
Sine 1D
Number of evaluations
Optimum
PROTOCOL
EI
PI
UCB
Random
Hartmann 3D
Objective value
Number of evaluations
Optimum
PROTOCOL
EI
PI
UCB
Random
Hartmann 6D
Objective value
Number of evaluations

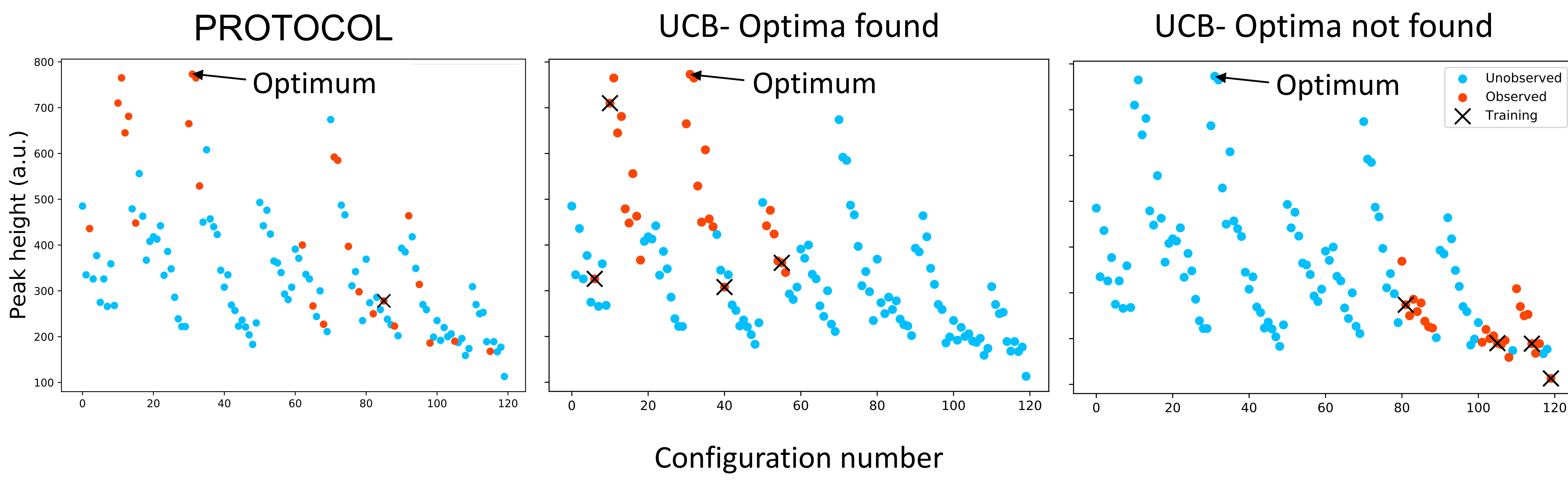
PROTOCOL
UCB- Optima found
UCB- Optima not found
Optimum
Optimum
Optimum
Unobserved
Observed
Training
Peak height (a.u.)
Configuration number
800
700
600
500
400
300
200
100
0
20
40
60
80
100
120
