# Supplementary material for: Asynchronous parallel Bayesian optimization for AI-driven cloud laboratories
Source: Bioinformatics. 2021 Jul 12;37(Suppl 1):i451–9. doi: 10.1093/bioinformatics/btab291 (PMC8275326; doi:10.1093/bioinformatics/btab291)
Supplement: btab291_Supplementary_Data [file btab291_supplementary_data.zip › btab291-suppl_data/Frisby.78.sup.5.pdf]

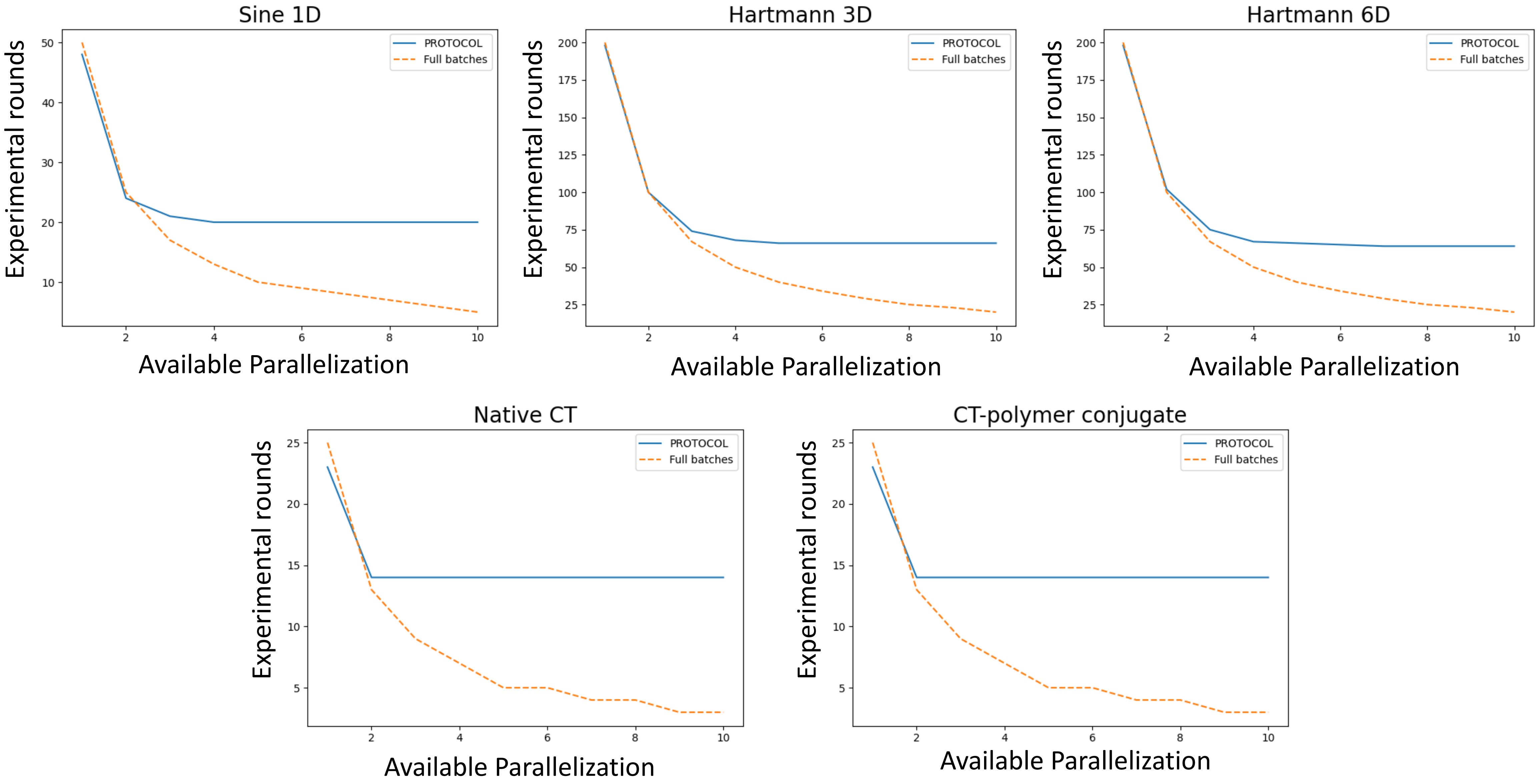
Sine 1D
Hartmann 3D
Hartmann 6D
Native CT
CT-polymer conjugate
PROTOCOL
Full batches
Experimental rounds
Available Parallelization

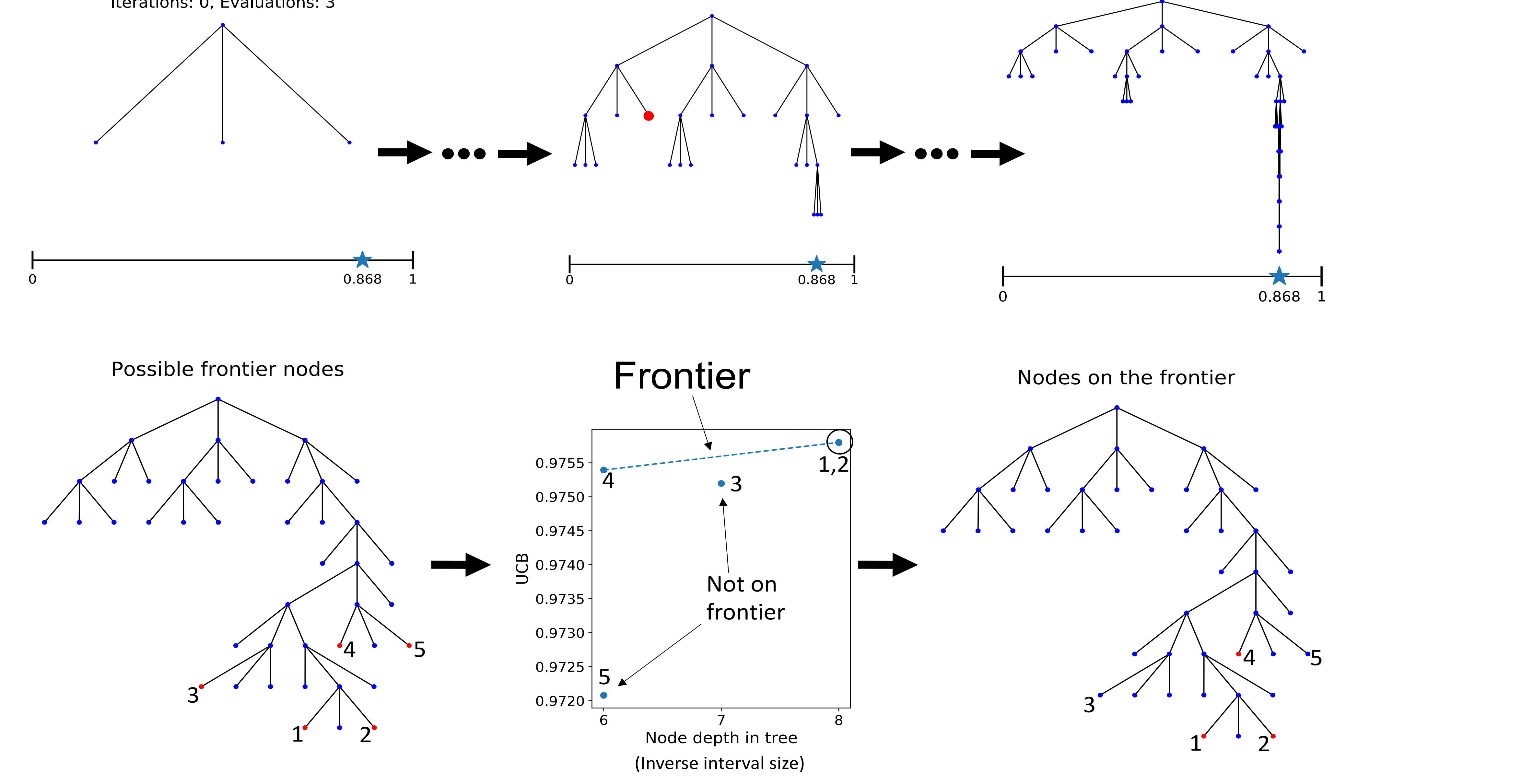

Iterations: 0, Evaluations: 3
0
0.868
1
Possible frontier nodes
1
2
3
4
5
Frontier
UCB
0.9755
0.9750
0.9745
0.9740
0.9735
0.9730
0.9725
0.9720
6
7
8
Node depth in tree
(Inverse interval size)
1,2
Not on
frontier
Nodes on the frontier

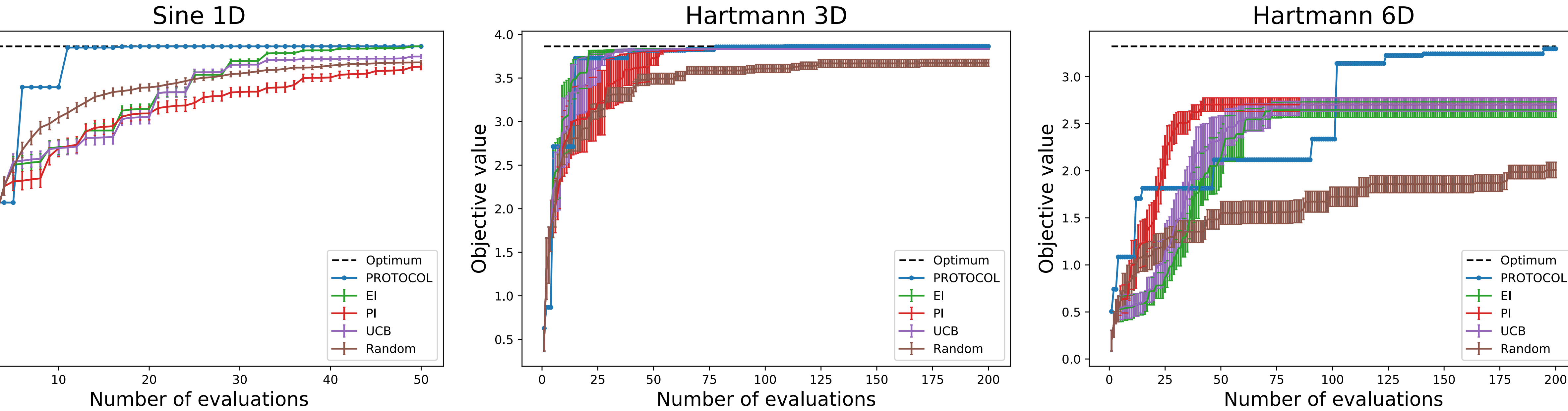

Sine 1D
Hartmann 3D
Hartmann 6D
Objective value
Number of evaluations
Optimum
PROTOCOL
EI
PI
UCB
Random

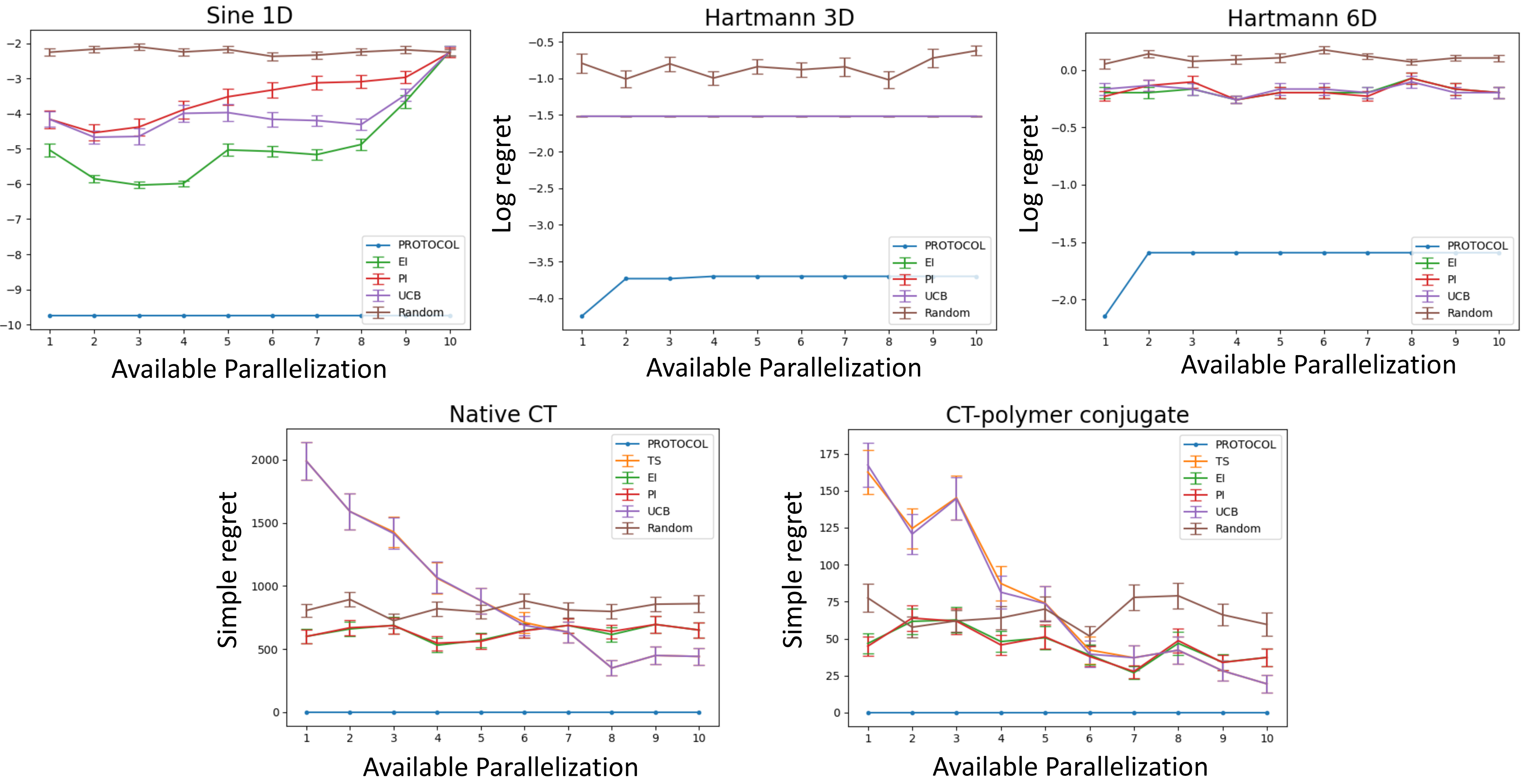

Sine 1D
Hartmann 3D
Hartmann 6D
Native CT
CT-polymer conjugate
Log regret
Simple regret
Available Parallelization
PROTOCOL
TS
EI
PI
UCB
Random

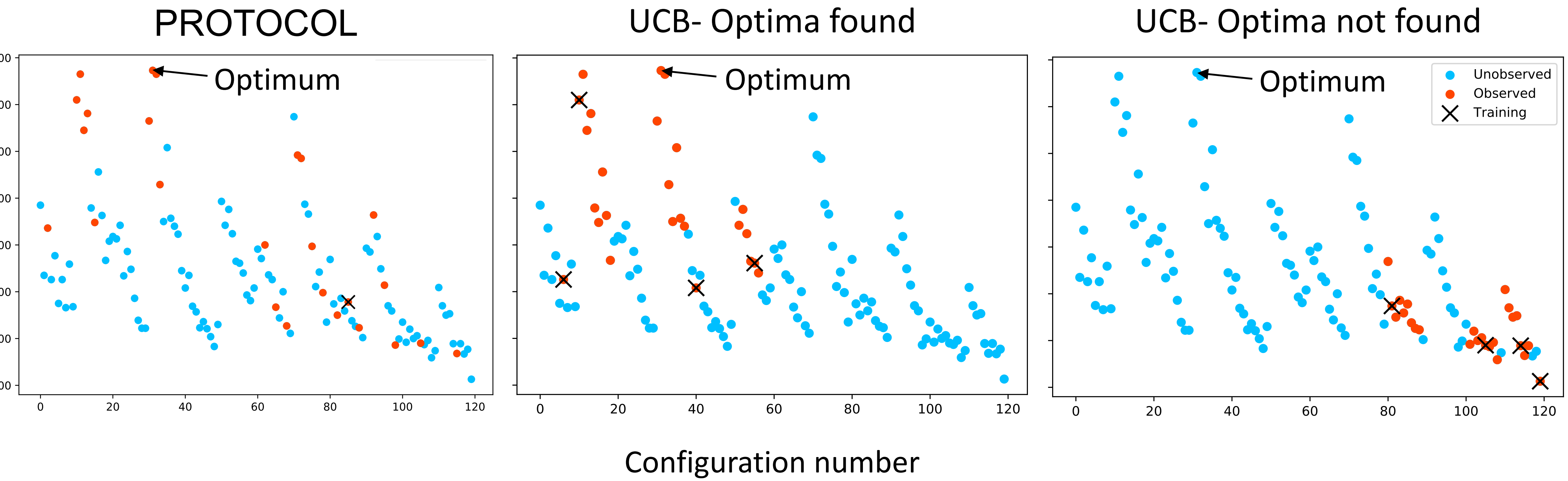

PROTOCOL
UCB- Optima found
UCB- Optima not found
Optimum
Optimum
Optimum
Unobserved
Observed
Training
0
20
40
60
80
100
120
Configuration number
