# Supplementary figures and images for: Asynchronous parallel Bayesian optimization for AI-driven cloud laboratories
Source: Bioinformatics. 2021 Jul 12;37(Suppl 1):i451–9. doi: 10.1093/bioinformatics/btab291 (PMC8275326; doi:10.1093/bioinformatics/btab291)

# 1D Sinusoidal Function

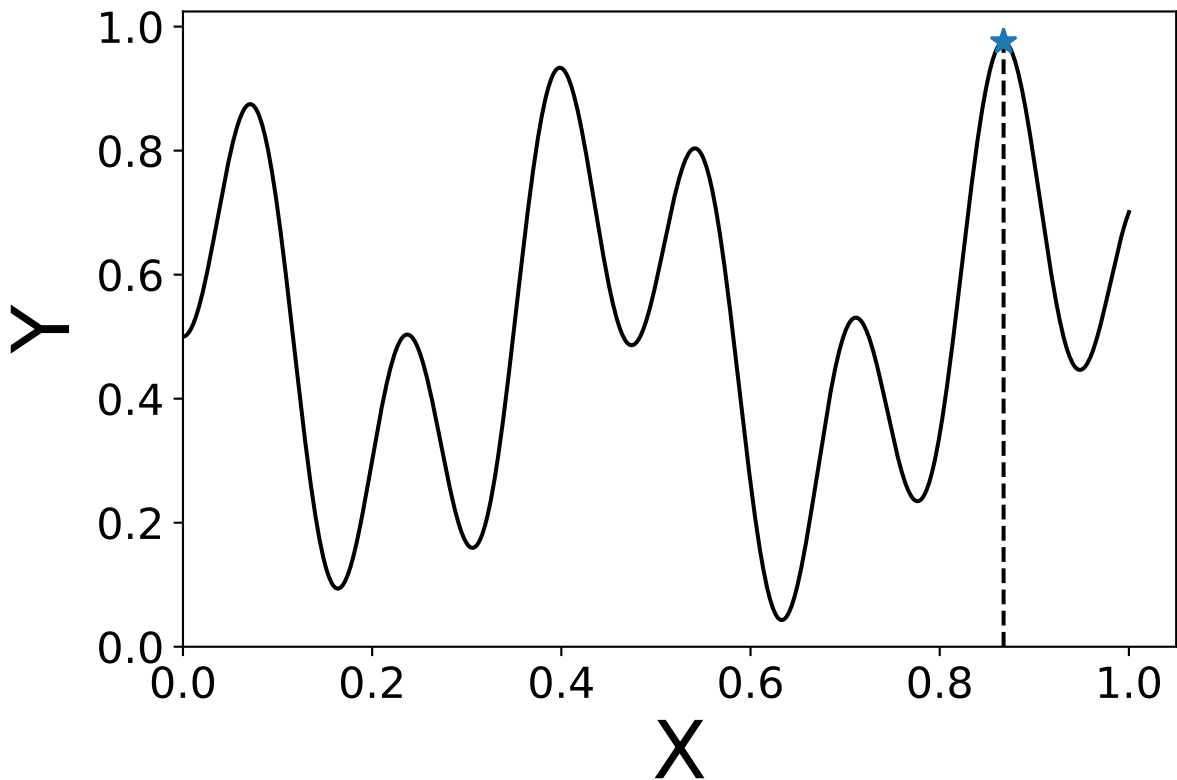

Supplement: btab291_Supplementary_Data [file btab291_supplementary_data.zip › btab291-suppl_data/Frisby.78.sup.2.pdf]
